# Neuroimaging of the joint Simon effect with believed biological and non-biological co-actors

**DOI:** 10.3389/fnhum.2015.00483

**Published:** 2015-09-01

**Authors:** Tanya Wen, Shulan Hsieh

**Affiliations:** ^1^Cognitive Electrophysiology Lab: Control, Aging, Sleep, and Emotion, Department of Psychology, National Cheng Kung UniversityTainan, Taiwan; ^2^Department of Life Sciences, National Cheng Kung UniversityTainan, Taiwan; ^3^Institute of Allied Health Sciences, National Cheng Kung UniversityTainan, Taiwan; ^4^Department of Public Health, National Cheng Kung UniversityTainan, Taiwan

**Keywords:** joint Simon task, Go/Nogo, biological co-actor, action representation, medial prefrontal cortex

## Abstract

Performing a task alone or together with another agent can produce different outcomes. The current study used event-related functional magnetic resonance imaging (fMRI) to investigate the neural underpinnings when participants performed a Go/Nogo task alone or complementarily with another co-actor (unseen), whom was believed to be another human or a computer. During both complementary tasks, reaction time data suggested that participants integrated the potential action of their co-actor in their own action planning. Compared to the single-actor task, increased parietal and precentral activity during complementary tasks as shown in the fMRI data further suggested representation of the co-actor’s response. The superior frontal gyrus of the medial prefrontal cortex was differentially activated in the human co-actor condition compared to the computer co-actor condition. The medial prefrontal cortex, involved thinking about the beliefs and intentions of other people, possibly reflects a social-cognitive aspect or self-other discrimination during the joint task when believing a biological co-actor is present. Our results suggest that action co-representation can occur even oﬄine with any agent type given *a priori* information that they are co-acting; however, additional regions are recruited when participants believe they are task-sharing with another human.

## Introduction

The Simon effect (Simon and Small, 1969) is a well-known phenomenon, in which participants carry out responses that are either congruently or incongruently matched with the stimuli (see Simon and Berbaum, 1990; Lu and Proctor, 1995 for reviews). For example, a common protocol for a Simon task is to ask participants to carry out button presses with either their left or right hand in response to stimuli that randomly appear on the left or right of the screen (e.g., use left hand to respond to green circles and right hand to respond to red circles). Even though the location of the stimulus is task-irrelevant, responses are typically faster when they are spatially congruent with the hand used to respond. This effect disappears if participants are asked to perform with one response key, i.e., in a Go/Nogo task (Sebanz et al., 2003; Dolk et al., 2014; Pfister et al., 2014).

The joint Simon task, also known as the social Simon task (Sebanz et al., 2003), is when two participants perform the Simon task together, each doing half of the task, in other words, they are doing complementary Go/Nogo tasks. This time each participant only uses one hand to respond to stimuli that randomly appear on the left or right of the screen (e.g., one participant respond to green circles and the other participant respond to red circles). However, this also produces a Simon effect: the participant positioned on the right responds faster to stimuli on the right of the screen (and vice versa for the participant sitting on the left). Thus it is known as the joint Simon effect.

In the history of studying the joint Simon effect, there has been a number of theories proposed aiming to explain its mechanism. Some theories emphasize “social” mechanisms (Knoblich and Sebanz, 2006; Sebanz et al., 2006a; Tsai et al., 2006; Tsai and Brass, 2007; Sebanz and Knoblich, 2009), when performing the task together, the joint Simon effect is caused by the integration of the other person or the other person’s action into one’s own action planning, task representation, or body representation (i.e., action co-representation, Sebanz et al., 2003, 2006a; Wenke et al., 2011). On the other hand, according to the spatial response coding account (Guagnano et al., 2010; Dittrich et al., 2012, 2013) the joint Simon effect occurs because the co-actor or attention-attracting objects provides a reference for the participant to code their actions spatially. However, both social and spatial response coding accounts fail to explain a number of observations. For example, it has been demonstrated that the knowledge about the co-actor’s task is neither necessary nor sufficient for the Simon effect to occur (Dolk et al., 2013a). Through a series of behavioral experiments, Dolk et al. (2011) demonstrated reliable joint Simon effects when the co-actor was not actively involved and even when the co-actor was absent; in another study, Dolk et al. (2013a) have successfully shown that non-biological objects, such as Japanese waving cat, a clock, or a metronome can generate joint Simon effect. Recently, building on the theory of event coding (Hommel, 2009), which is derived from earlier ideomotor and common coding frameworks (Prinz, 1984, 1997), Dolk et al. (2014) suggested a more comprehensive referential coding account that integrates aspects of previous theories; social and spatial response coding. According to this account, response conflict occurs when activation of multiple action representations are activated at the same time, and referential coding is required to distinguish between concurrently activated salient events. As the similarity of action events increases, the difficulty to discriminate between alternative codes is greater, leading to larger Simon effects.

The joint Simon effect has been studied mostly using behavioral (e.g., Colzato et al., 2012a,b; Liepelt et al., 2013; Sellaro et al., 2015) or ERP measures (e.g., Sebanz et al., 2006b; Tsai et al., 2006; de Bruijn et al., 2008); however, due to the several technical restraints of the functional magnetic resonance imaging (fMRI) scanner, such as huge machinery dimensions, loud noise, and horizontal lying position influencing the social dimension of experimental settings (Costantini et al., 2013), there lacks sufficient studies on the neural correlates of the joint Simon effect. To our knowledge, only one experiment using fMRI has been published to investigate the joint Simon effect (Sebanz et al., 2007). In the fMRI study by Sebanz et al. (2007), a confederate entered the fMRI scanning room with the participant to carry out the joint Simon task. By contrasting joint Simon task and Go/Nogo task, several activated regions were identified. Using Nogo trials as a baseline, Go trails showed increased activation in the medial frontal cortex, anterior cingulate gyrus, and frontal eye fields, which are possibly related to metacognition and self-relevance. During Nogo trials (with Go trials as a baseline), when it was the confederate’s turn, the parietal lobe and supplementary motor area were more activated, which reflected increased inhibition to refrain from acting when it was the other’s turn. These brain patterns likely reflect a social cognitive-aspect of the joint Simon task. However, in order to conquer the technical restraints, Sebanz et al. (2007) used an intricate apparatus setup, in which a confederate acted with the participant using a response box placed upon the participant’s belly, and a set of mirrors was positioned on the head coil so that the participant could see their own hand and the hand of the co-actor. With this kind of experimental setting, the joint Simon effect that Sebanz et al. (2007) observed could be attributed to either the social or the spatial response coding factor. Hence, the mechanism of the joint Simon effect remained equivocal.

Therefore, given that there were very few (and perhaps only one) fMRI studies and inspired by some behavioral and ERP studies that successfully induced the joint Simon effect with an unseen co-actor (e.g., Tsai et al., 2008; Vlainic et al., 2010; Dolk et al., 2013b), the current experiment aimed to re-investigate the joint Simon effect by using event-related fMRI and adopted a pure belief paradigm as in Tsai et al.’s (2008) ERP study, in which the participant performed the joint Simon task with a believed human co-actor or a computer co-actor located outside the scanning room. A solo Go/Nogo task served as a control. The aims of the current study are to examine three main questions: (1) what are the neural underpinnings of the joint Simon effect? The standard Simon task draws on inhibitory control to resolve response conflict of multiple concurrently activated responses. fMRI studies have found that these tasks activate the fronto-parietal regions including the anterior cingulate cortex, dorsolateral prefrontal cortex, inferior frontal gyrus, posterior parietal cortex, and anterior insula (Nee et al., 2007; Schumacher et al., 2007) as well as visuospatial and visual attention processing areas (Liu et al., 2004). Given that the behavioral Simon effect of joint Simon tasks, we expect to see similar activations in these areas in conditions where there is a significant joint Simon effect. (2) We want to address whether joint Simon effects can be observed when the participants are told that the co-actor is a computer. If social factors are essential to generate the joint Simon effect, then we would expect to observe the effect only in the belief of biological co-actor condition, and not in the non-human co-actor condition (as in Tsai and Brass, 2007; Tsai et al., 2008). On the other hand, if spatial response coding is essential for the joint Simon effect, then we would probably not be able to observe any joint Simon effect in the current experimental setting. However, according to theories derived from ideomotor theories, such as theory of event coding or the referential coding account, the presence of another co-acting agent could produce the joint Simon effect, therefore, it is possible to observe joint Simon effects in both biological and computer co-actors. (3) The last question is whether there are differences between the participants’ belief of co-actor agent. The medial prefrontal cortex has been implicated as a region for social cognition, and is activated when thinking about the self and others (Amodio and Frith, 2006). Sebanz et al. (2007) found increased orbitofrontal cortex activation, a part of the medial frontal cortex, when co-acting with another person in the joint Simon task compared to the single actor condition. We further hypothesize that the medial prefrontal cortex activity would be higher in the biological co-actor computer co-actor condition if social cognitive processes are involved.

## Materials and Methods

### Participants

Thirty-six healthy participants (18 males and 18 females) from southern Taiwan participated in the experiment (age range = 20–30 years, mean age = 22.25 years, *SD* = 2.05 years). The participants were right-handed (indicated by the Edinburgh Handedness Inventory), had normal or corrected-to-normal vision, and no history of psychological or neural disorders. Their BDI, BAI, and IQ scores were in the normal range (BDI: 0–10; BAI: 0–8; Raven’s Standard Progressive Matrices test score: 34–59). All participants provided their written informed consent, and the study protocol was approved (NO: B-ER-101-144) by the Institutional Review Board (IRB) of the National Cheng Kung University Hospital, Tainan, Taiwan. All participants were paid 600 NTD after completion of the experiment.

### Experimental Setting and Design

We manipulated four different conditions: (1) believed biological agent co-actor joint Simon task, (2) computer co-actor joint Simon task, (3) single Go/Nogo task, and (4) standard Simon task. The first three conditions were presented in a random order between participants (perfectly counterbalanced using a 3 × 3 Latin square), while the standard Simon task was always situated last to minimize task interference, such as carryover effects (Ansorge and Wuhr, 2004, 2009; Lugli et al., 2013).

At the beginning of experiment, participants were acquainted with a confederate who pretended to be another participant participating in the experiment. The participant and confederate wrote questionnaires together, and practiced performing a joint Simon task together (16 trials in total). During the practice, the participant always sat to the right of the confederate, while a colored circle target (either red or green) would appear on either the right or left side. Each were assigned to respond to a specific color (which was counterbalanced between participants), with the participant using his/her right hand to press “9” (located right on the keyboard) and the confederate using his/her right hand to press “4” (located left on the keyboard) when each person’s assigned color was detected.

The participant was told that he/she would do the task in the fMRI scanner, while his/her partner was signed up to participate in the co-acting behavioral task outside. To reinforce the belief of interacting with another participant, before the believed biological agent co-actor condition, the participant and the confederate were allowed to communicate through an intercom system. In reality, however, the responses were controlled by a computer (the response time varying randomly from 300 to 450 ms). Once a response was made, the stimuli are removed from the screen.

In the computer co-actor joint Simon task, participants were told to respond to one color, and that the computer responded to the other color (the response time varied randomly from 300 to 450 ms). In the single Go/Nogo task, participants were told that they were to carry out this task alone; they were asked to respond to the target color, and to inhibit responding to the non-target color. Lastly, in the standard Simon task, participants responded to one color with the right hand, and to the other color with the left hand. However, due to the absence of Nogo trials in the standard Simon task, only behavioral results of the standard Simon task are reported.

### Stimuli and Procedure

Stimuli presentation was rear-projected onto a screen inside the magnetic resonance imaging (MRI) scanner that was 95 cm away from the observer. Participants viewed the display through a mirror that was placed above the head coil. The visual stimuli consisted of two circles (with ∼2.5 cm radius and 5 cm between the disks) horizontally placed within a white rectangle frame (∼15 cm × 5 cm in width and height). In each trial, one of the circles will be colored either green or red (serving as a target), and the remaining circle will be white. Each circle extended ∼3° from the center. Participants held two Current Designs fiber optic response pads, one in each hand. The response pads each have four buttons from top to down in vertical view; and participants were instructed at the beginning to hold the response pad vertically and response using only the topmost button (and ignore all the other buttons).

At the beginning of each trial, a fixation cross was presented for 500 ms. This was followed by the target which was displayed on the screen up to 1400 ms or a response was given. Participants were instructed to respond to their assigned color by pressing a button on a response pad. In joint tasks (biological and computer co-actor conditions), the responses made by the computer were randomized within 300–450 ms to make it seem as if another person was responding. Afterward, a blank screen (lasting from 0 to 8 s) followed before the start of the next trial.

Each condition constituted a separate run (therefore four runs in total). Each run consisted of 160 trials (with 40 trials for each trial type: Go-congruent, Go-incongruent, Nogo-congruent, Nogo-incongruent), lasting ∼9.5 min. The order and the timing of each trial was pseudorandomized using optseq2 (Dale, 1999) to provide an optimum jittered sequence. Stimulus presentation was controlled using E-PRIME 2.0 software (Psychology Software Tools, Pittsburgh, PA, USA).

### Behavioral Analyses

Error trials and outliers (greater than three interquartile range from the mean) were removed from analysis. A repeated-measure 2 × 4 ANOVA with factors condition (believed biological agent co-actor joint Simon task, computer co-actor joint Simon task, single Go/Nogo task, and standard Simon task) and congruency (congruent versus incongruent) was conducted.

### Imaging Parameters and Data Analyses

Imaging was performed using the GE MR750 3T scanner (GE Medical Systems, Waukesha, WI, USA) in the MRI center of National Cheng Kung University. High resolution anatomical images were acquired using fast-SPGR, consisting of 166 axial slices (TR = 7.6 ms, TE = 3.3 ms, flip angle = 12°, 224 × 224 matrices, slice thickness = 1 mm). Functional images were acquired using a gradient-echo echo-planar imaging (EPI) pulse sequence (TR = 2000 ms, TE = 33 ms, flip angle = 90°, 64 × 64 matrices, slice thickness = 3 mm, no gap, voxel size 3.5 mm × 3.5 mm × 3 mm, 40 axial slices covering the entire brain).

The data was preprocessed and analyzed using BrainVoyager QX (Brain Innovation, Maastricht, The Netherlands) and customized Matlab scripts (2010a, The MathWorks, Inc., Natick, MA, USA). Functional images were corrected for head movements using six-parameter rigid transformations, after slice timing correction, by realigning all volumes to the first functional volume. High-pass filtering of two cycles and spatial smoothing of 4 mm FWHM were applied. For each participant, the functional scan was co-registered to the anatomical scan and then transformed into Talairach space (Talairach and Tournoux, 1988).

Statistical analyses were performed first at the individual level using general linear modeling (GLM). Incorrect trials and outliers were not modeled; reaction times were modeled. Contrast images for each participant were then subjected to a random effects group analysis to identify common brain regions across participants that show main effects and interactions between response and congruency. All statistical thresholds were corrected for multiple comparisons using the alphasim command in Matlab, Neuroelf (http://neuroelf.net/) to keep the familywise error rate under 5%, and the corrected threshold was set at *p* < 0.005 and cluster size >20 voxels. Visualization was also aided by Neuroelf.

## Results

### Behavioral Results

#### Overall Behavioral Data Summary

The overall accuracy was high (98.88% across all conditions), erroneous trials were eliminated from analysis (error rates of each condition were 0.6, 0.3, 0.4, and 2.6% for the biological co-actor, computer co-actor, single Go/Nogo, and standard Simon conditions, respectively). 1.98% of the remaining go trials were classified as outliers and thus not considered. To compare performance between the four conditions, a condition (biological co-actor, computer co-actor, single Go/Nogo, and right hand of standard Simon) × congruency (congruent versus incongruent) ANOVA was conducted. There were significant main effects for both condition [*F*(3,105) = 116.18, *p* < 0.001] and congruency [*F*(1,35) = 31.88, *p* < 0.001]. The interaction between condition and congruency was also significant [*F*(3,105) = 15.45, *p* < 0.001]. *Post hoc* analysis showed that the reaction times during the Standard Simon condition task was longer than all three conditions (all *t*s > 11.79, all *p*s < 0.001); there were no differences among the remaining three conditions (all *t*s < 0.93, all *p*s > 0.36).

**FIGURE 1:**
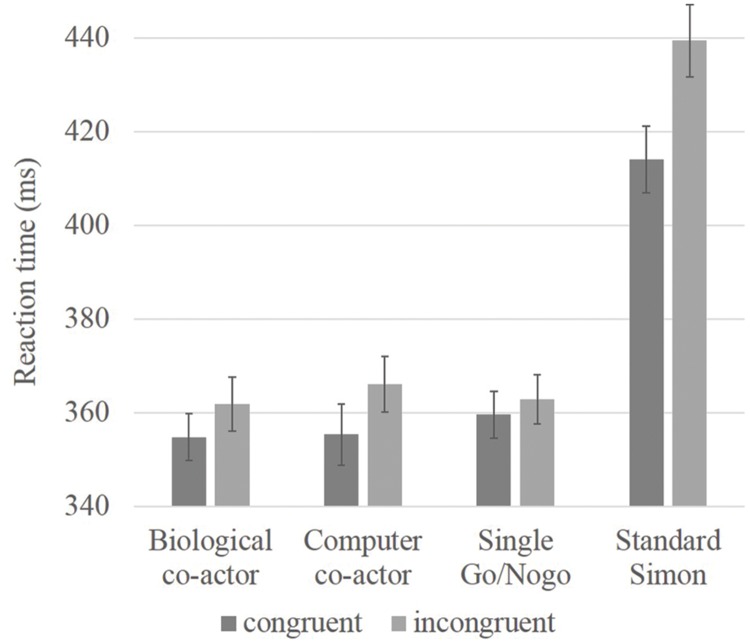
**Bar graph showing reaction time data of congruency effects in believed biological co-actor, computer co-actor, single Go/Nogo, and standard Simon conditions.** Reaction times were slower in the standard Simon task. Significant differences between incongruent and congruent conditions were observed in the believed biological co-actor and computer co-actor conditions, but not in the single Go/Nogo condition. Error bars depict the standard error.

Given that the Simon effect in the standard Simon condition is much larger than the effects in the other conditions, the inclusion of these data may be sufficient to drive the main effect of congruency and the interaction with condition, a separate 3 × 2 repeated measures ANOVA was conducted with only the three single hand conditions (biological co-actor, computer co-actor, single Go/Nogo). There were no main effects for condition [*F*(2,70) = 0.49, *p* = 0.62], there was a main effect for congruency [*F*(1,35) = 10.20, *p* = 0.003], and a near significant interaction between condition and congruency [*F*(2,70) = 3.00, *p* = 0.056]. Next, *post hoc* analyses were performed; simple main effects of the condition × congruency interaction showed a significant difference between incongruent and congruent trials in the biological co-actor, and computer co-actor tasks [*F*(1,105) = 6.34, *p* = 0.01; *F*(1,105) = 14.59, *p* < 0.001], but no congruency effects in the single Go/Nogo task [*F*(1,105) = 1.42, *p* = 0.24]. Results are shown in **Figure 1**.

### fMRI Results

#### ANOVA: Main Effects and Interactions

**FIGURE 2:**
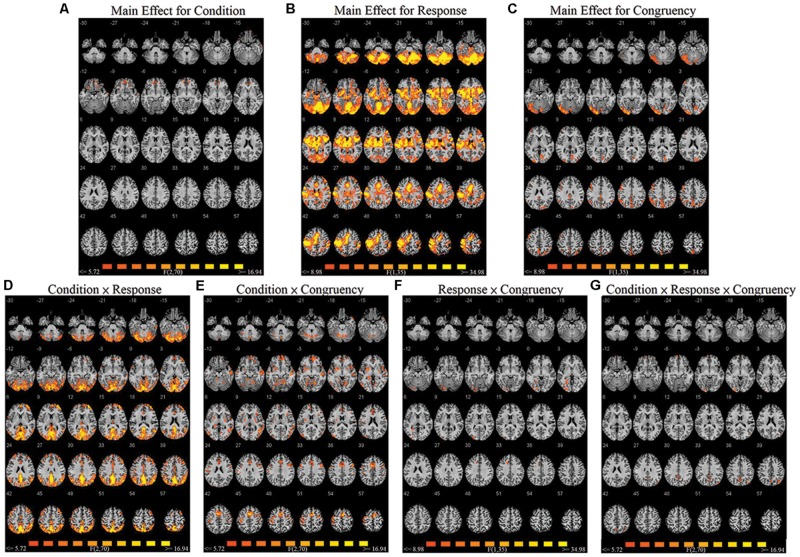
**Montage of transversal slicing (the numbers indicate the z coordinates) of the ANOVA results of the joint Simon task, with factors condition (biological co-actor, computer co-actor, and single Go/Nogo), response (Go versus Nogo), and congruency (congruent versus incongruent). (A)** main effect for condition, **(B)** main effect for response, **(C)** main effect for congruency, **(D)** two-way interaction of condition and response, **(E)** two-way interaction of condition and congruency, **(F)** two-way interaction of response and congruency, and **(G)** three-way interaction of condition, response, and congruency.

A repeated-measures three-way ANOVA was performed at the whole-brain level, with factors condition (biological co-actor, computer co-actor, single Go/Nogo), response (Go versus Nogo), and congruency (congruent versus incongruent). There were significant main effects and interactions in various brain regions. **Figure 2** and **Table 1** depict these results. In order to investigate which levels drove the main effects and interactions, we followed-up the ANOVA results with *post hoc t*-tests using the contrasts in Sebanz et al. (2007).

**Table 1:** Brain activation data of ANOVA results, with factors condition (biological co-actor, computer co-actor, and single Go/Nogo), response (Go versus Nogo), and congruency (congruent versus incongruent).

	# of voxels	Talariach coordinates (peak)		
Structure	3^∗^3^∗^3	*x*	*y*	*z*
**Main effects**				
**Main effect for condition**				
B Anterior cingulate (including anterior cingulate, medial frontal gyrus, superior frontal gyrus)	129	9	41	1
**Main effect for response**				
B Culmen (including culmen, thalamus, declive, caudate, insula, culmen of vermis, inferior parietal lobule, post-central gyrus, lentiform nucleus, cingulate gyrus, precentral gyrus, medial frontal gyrus, fusiform gyrus, inferior frontal gyrus, inferior occipital gyrus, lingual gyrus, anterior cingulate, declive of vermis, middle occipital gyrus, sub-gyral, claustrum, inferior temporal gyrus, pyramis, midbrain red nucleus, middle temporal gyrus, superior parietal lobule, cuneus, middle frontal gyrus, superior frontal gyrus, posterior cingulate, parahippocampal gyrus, supramarginal gyrus, tuber, cerebellar lingual, superior temporal gyrus, uvula, middle temporal gyrus, precuneus)	16984	21	-49	-20
L Cingulate gyrus	174	0	-55	31
R Post-central gyrus (including post-central gyrus, precentral gyrus)	129	36	-25	52
L Angular gyrus (including angular gyrus, middle temporal gyrus, superior temporal gyrus)	103	-45	-70	34
L Middle frontal gyrus	66	-36	44	28
R Middle temporal gyrus	44	45	-55	28
**Main effect for congruency**				
B Inferior temporal gyrus (including inferior temporal gyrus, middle occipital gyrus, lingual gyrus, cuneus, fusiform gyrus, inferior occipital gyrus, precuneus, cingulate gyrus, declive, paracentral lobule, sub-gyral)	1308	-48	-73	-8
L Precentral gyrus (including precentral gyrus, middle frontal gyrus, inferior frontal gyrus)	115	-45	-1	28
L Inferior frontal gyrus	27	-39	47	4
R Precuneus	61	27	-70	46
L Inferior parietal lobule (including inferior parietal lobule, superior temporal gyrus, angular gyrus, supramarginal gyrus)	154	-36	-55	52
R Supramarginal gyrus	47	51	-46	37
R Middle occipital gyrus	38	27	-91	13
**Two-way interactions**				
**Condition × Response**				
B Cuneus (including cuneus, cingulate gyrus, precuneus, lingual gyrus, middle occipital gyrus, posterior cingulate, occipital lobe Brodmann area 19, paracentral lobule, superior parietal lobule, middle temporal gyrus, inferior parietal lobule, fusiform gyrus, declive, inferior temporal gyrus, superior temporal gyrus, thalamus, medial frontal gyrus, angular gyrus, supramarginal gyrus, sub-gyral, culmen of vermis, anterior cingulate, post-central gyrus, precentral gyrus, inferior occipital gyrus)	7586	0	-64	40
B Middle frontal gyrus (including middle frontal gyrus, superior frontal gyrus, medial frontal gyrus, inferior frontal gyrus)	716	36	53	10
L Precentral gyrus (including precentral gyrus, superior frontal gyrus, middle frontal gyrus)	211	-45	2	43
R Middle temporal gyrus	44	54	-28	-17
L Precentral gyrus	23	-60	8	7
**Condition × Congruency**				
B Cingulate gyrus (including cingulate gyrus, medial frontal gyrus, superior frontal gyrus, cingulate gyrus, sub-gyral, anterior cingulate, middle frontal)	690	0	2	46
R Middle temporal gyrus (including middle temporal gyrus, middle occipital gyrus, superior temporal gyrus)	130	45	-58	10
B Declive (including declive, lingual gyrus, culmen of vermis)	328	-12	-67	-17
L Post-central gyrus (post-central gyrus, inferior parietal lobule, precentral gyrus)	197	-33	-22	46
L Lentiform nucleus	123	-27	2	1
R Precentral gyrus (including precentral gyrus, inferior frontal gyrus)	153	48	5	37
R Superior temporal gyrus cluster 1	109	45	-19	-5
R Superior temporal gyrus cluster 2	24	51	14	-14
L Middle occipital gyrus cluster 1 (including middle occipital gyrus, middle temporal gyrus)	52	-33	-85	19
L Middle occipital gyrus cluster 2	24	-30	88	-2
L Middle temporal gyrus	57	-51	-67	4
L Lingual gyrus (including lingual gyrus, thalamus)	64	-15	-43	-8
R Post-central gyrus	26	45	-25	49
R Insula	76	45	14	1
L Insula cluster 1 (including inferior parietal lobule)	45	-42	-22	25
L Insula cluster 2	30	-30	20	10
L Superior temporal gyrus	26	-57	-10	1
R Lentiform nucleus	37	24	-7	7
L Culmen	21	-33	-52	-20
R Superior temporal gyrus	24	54	-4	4
L Precentral gyrus	39	-45	-4	25
**Response × Congruency**				
L Middle occipital gyrus	115	-24	-88	4
L Cingulate gyrus	21	-15	20	31
L Parahippocampal gyrus	34	-21	-46	4
**Three-way interactions**				
**Condition × Reponse × Congruency**				
L Middle occipital gyrus	25	-27	-88	1
R Precuneus	52	36	-70	40
L Medial frontal gyrus	20	-6	68	-2
B Cingulate gyrus (including cingulate gyrus, cuneus)	57	-3	-43	31

*^∗^B, L, and R denote bilateral, left, and right, respectively. All regions reached a significance threshold of *p* < 0.005 (uncorrected), and voxel threshold of at least 20 contiguous voxels.*

#### Effects of Co-action on Go Trials

**FIGURE 3:**
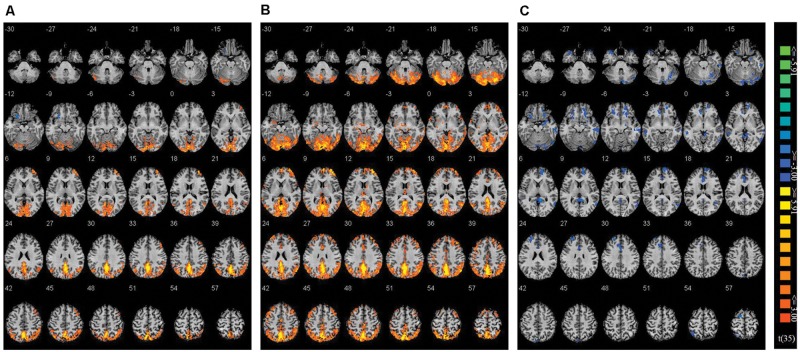
**Montage of transversal slicing (the numbers indicate the z coordinates) of using Nogo trials as a baseline and comparing the activity of Go trials in **(A)** biological co-actor condition versus the single-actor No/Nogo condition; **(B)** computer co-actor condition versus the single-actor No/Nogo condition; and **(C)** biological co-actor condition versus the computer co-actor condition**.

Using Nogo trials as a baseline, we respectively compared activity on Go trials among the two co-actor conditions (believed biological agent co-actor and computer co-actor) and the single Go/Nogo tasks (i.e., Go-congruent + Go-incongruent > Nogo-congruent + Nogo-incongruent). The contrasts are depicted in **Figure 3**. Peak coordinates of the ROIs are listed in **Table 2**. These contrasts indicated that the biological co-actor condition compared to the single Go/Nogo condition showed higher activation in the cingulate gyrus, posterior cingulate, cuneus, precuneus, inferior parietal lobule, lingual gyrus, middle occipital gyrus, superior occipital lobule, middle temporal gyrus, supramarginal gyrus, fusiform gyrus, declive, middle frontal gyrus, and superior frontal gyrus, and less activation in the insula. The computer co-actor condition compared to the single Go/Nogo condition showed higher activation in the same regions, with additional regions including the anterior cingulate, superior parietal lobule, occipital lobe Brodmann area 19, superior occipital gyrus, inferior occipital gyrus, superior temporal gyrus, inferior temporal gyrus, parahippocampal gyrus, angular gyrus, precentral gyrus, post-central gryus, paracentral lobule, medial frontal gyrus, inferior frontal gyrus, thalamus, culmen, culmen of vermis, pyramis, lentiform nucleus, claustrum, but no differences in the superior occipital lobule and insula. Significant differences between the biological and computer co-actor conditions occurred in the anterior cingulate, posterior cingulate, cingulate gyrus, precuneus, superior parietal lobule, middle occipital gyrus, middle temporal gyrus, middle frontal gyrus, medial frontal gyrus, inferior frontal gyrus, lentiform nucleus, sub-gyral, extra-nuclear, and culmen, declive.

**Table 2:** Brain activation data.

		Direction of activation	# of voxels	Talariach coordinates (peak)		
Structure			3^∗^3^∗^3	*x*	*y*	*z*
**Contrast**	**Biological co-actor versus single Go/Nogo**					
	**Go – Nogo**					
	B Cuneus (including cuneus, cingulate gyrus, precuneus, posterior cingulate, lingual gyrus, middle occipital gyrus, middle temporal gyrus, inferior parietal lobule, supramarginal gyrus, fusiform gyrus, declive, superior occipital lobule)	+	2816	0	-64	40
	R Middle frontal gyrus cluster 1 (including middle frontal gyrus, superior frontal gyrus)	+	101	36	53	10
	R Middle frontal gyrus cluster 2	+	63	33	32	37
	R Insula	-	21	24	23	-8
	**Computer co-actor versus single Go/Nogo**					
	**Go – Nogo**					
	B Cuneus (including cuneus, cingulate gyrus, occipital lobe Brodmann area 19, precuneus, lingual gyrus, declive, posterior cingulate, fusiform gyrus, middle temporal gyrus, precentral gyrus, inferior temporal gyrus, angular gyrus, middle occipital gyrus, medial frontal gyrus, superior temporal gyrus, paracentral lobule, inferior occipital gyrus, culmen, superior parietal lobule, inferior parietal lobule, supramarginal gyrus, superior occipital gyrus, parahippocampal gyrus, culmen of vermis, post-central gyrus, pyramis, thalamus, anterior cingulate)	+	7495	-3	-64	37
	R Middle frontal gyrus (including middle frontal gyrus, medial frontal gryus, superior frontal gyrus, inferior frontal gyrus, precentral gyrus)	+	703	36	53	10
	L Superior frontal gryus	+	28	-18	17	55
	L Precentral gryus cluster 1 (including precentral gyrus, superior frontal gyrus, middle frontal gyrus)	+	262	-45	2	43
	L Precentral gryus cluster 2	+	29	-54	14	1
	L Post-central gyrus	+	78	-48	-16	46
	L Superior temporal gyrus	+	22	-63	-40	7
	R Precentral gyrus	+	20	48	14	1
	R Post-central gyrus	+	23	51	-19	19
	L Lentiform nucleus	+	26	-12	2	1
	L Claustrum	+	35	-30	5	-2
	R Thalamus	+	20	9	-13	13
	**Biological co-actor versus computer co-actor**					
	**Go–Nogo**					
	B Posterior cingulate (including posterior cingulate, culmen)	-	83	12	-43	7
	R Middle temporal gyrus cluster 1	-	70	60	-25	-20
	R Middle temporal gyrus cluster 2 (including middle temporal gyrus, middle occipital gyrus)	-	76	57	-55	10
	L Anterior cingulate cluster 1	-	24	-9	35	1
	L Anterior cingulate cluster 2	-	42	-3	26	19
	R Inferior frontal gyrus	-	38	18	26	-11
	R Middle frontal gyrus (including middle frontal gyrus, medial frontal gyrus)	-	129	15	44	-11
	B Precuneus	-	22	6	-46	64
	L Middle frontal gyrus	-	30	-24	47	25
	L Middle temporal gyrus	-	21	-57	-37	1
	L Cingulate gyrus	-	38	0	14	28
	R Lentiform nucleus	-	25	21	-4	-14
	L Superior parietal lobule	-	20	-33	-52	55
	L Sub-gyral	-	39	-18	5	58
	L Extra-nuclear	-	78	-24	20	-14
	R Declive	-	92	9	-82	-23

*Regions within significant clusters showing significant differences of activation on (1) Go trials in the believed biological co-actor condition compared to the single actor Go/Nogo condition; (2) Go trials in the believed computer co-actor condition compared to the single actor Go/Nogo condition; (3) Go trials in the believed biological co-actor condition compared to the believed computer co-actor condition.*

*^∗^B, L, and R denote bilateral, left, and right, respectively. All regions reached a significance threshold of *p* < 0.005 (uncorrected), and voxel threshold of at least 20 contiguous voxels.*

#### Congruency Effects on Go Trials

Congruency effects were calculated using Nogo trials as a baseline in the believed biological co-actor, computer co-actor, and single Go/Nogo conditions. We decided to implement the Nogo trials as a baseline given the following reasons: (1) the only other existing joint Simon fMRI study (Sebanz et al., 2007) also used Nogo trials as a baseline; (2) the Nogo-congruent trials to the participant would be the Go-incongruent trials to the co-actor, thus the contrast “Go-congruent – Nogo-congruent > Go-incongruent – Nogo-incongruent” would be equivalent to “Go-congruent (for participant) + Go-congruent (for co-actor) > Nogo-congruent (for participant) + Nogo-congruent (for co-actor),” and (3) this contrast would also parallel the standard Simon contrast “congruent (right hand) + congruent (left hand) > incongruent (right hand) + incongruent (left hand).”

**FIGURE 4:**
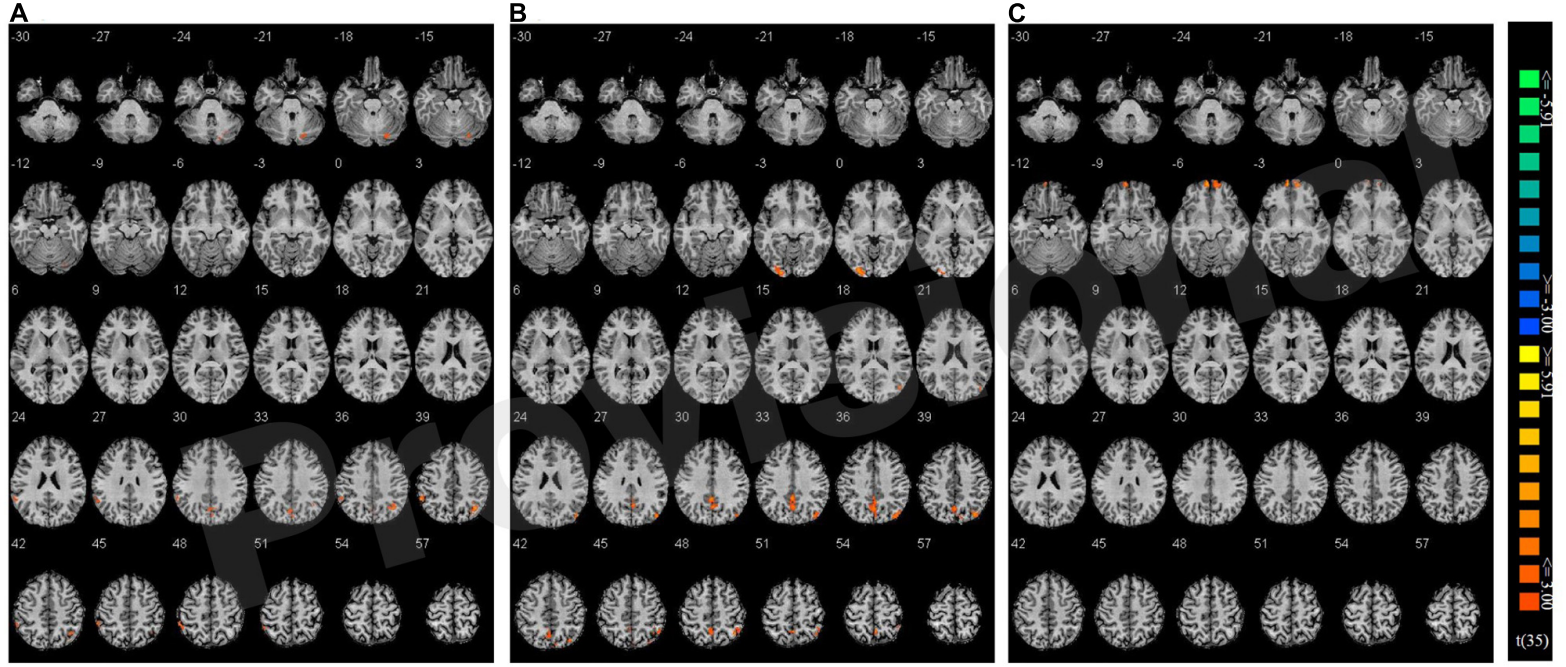
**Montage of transversal slicing (the numbers indicate the z coordinates) of using Nogo trials as a baseline and comparing the activity of congruent versus incongruent trials in **(A)** biological co-actor condition versus the single-actor No/Nogo condition; **(B)** computer co-actor condition versus the single-actor No/Nogo condition; and **(C)** biological co-actor condition versus the computer co-actor condition**.

Congruency effects were compared between the three single hand conditions (believed biological agent co-actor, computer co-actor, and single Go/Nogo task). The contrasts are shown in **Figure 4** and listed in **Table 3**. Compared to the single-actor condition, the biological co-actor condition elicited higher activation during congruent trials in the precuneus, cuneus, inferior parietal lobule, post-central gyrus, and fusiform gyrus. Compared to the single-actor condition, the computer co-actor condition elicited higher activation during congruent trials in the precuneus, inferior parietal lobule, and lingual gyrus. The difference between the biological and computer co-actor conditions in occurred in the bilateral superior frontal gyrus, which is a part of the medial prefrontal cortex.

**Table 3:** Brain activation data.

		Direction of activation	# of voxels	Talariach coordinates (peak)		
Structure			3^∗^3^∗^3	x	y	z
**Contrast**	**Biological co-actor versus single Go/Nogo**					
	**congruent – incongruent**					
	R Precuneus	+	32	36	-58	40
	L Inferior parietal lobule	+	42	-54	-40	49
	R Post-central gyrus	+	20	9	-31	73
	R Fusiform gyrus	+	24	27	-76	-17
	L Cuneus	+	21	-6	-67	34
	**Computer co-actor versus single Go/Nogo**					
	**congruent – incongruent**					
	L Lingual gyrus	+	35	-21	-91	-2
	R Inferior parietal lobule	+	22	39	-58	49
	R Precuneus	+	61	36	-70	40
	B Precuneus (including precuneus, cingulate gyrus, cuneus)	+	106	3	-52	55
	**Biological co-actor versus computer co-actor**					
	**congruent – incongruent**					
	L Superior frontal gyrus	+	32	-6	62	-5
	R Superior frontal gyrus	+	21	9	59	-5

*Using Nogo trials as a baseline, regions within significant clusters showing significant differences of activation on (1) congruent trials in the believed biological co-actor condition compared to the single actor Go/Nogo condition; (2) congruent trials in the believed computer co-actor condition compared to the single actor Go/Nogo condition; (3) congruent trials in the believed biological co-actor condition compared to the believed computer co-actor condition.*

*^∗^B, L, and R denote bilateral, left, and right, respectively. All regions reached a significance threshold of *p* < 0.005 (uncorrected), and voxel threshold of at least 20 contiguous voxels.*

## Discussion

In this experiment, Simon effects were observed both when the participants believed they were interacting with a human partner and while they were acting with a computer co-actor. No Simon effects were observed in the single Go/Nogo condition. In the literature, reports of the joint Simon effect appears to be very diverse. For example, some studies (Welsh et al., 2007; Sellaro et al., 2013) suggested that without another co-actor physically present (i.e., when they did the task through a networked computer in another room), belief alone of interacting with another agent is not sufficient to generate the joint Simon effect. However, believing was sufficient to activate the processes of response co-representation in Tsai et al.’s (2008) as well as in Ruys and Aarts (2010) study, in which only one participant performed the joint Simon task while believing they were interacting with the other, where in fact their partner’s response was generated by a computer. Although the experimenters found that while belief of a human agent was enough to result in a joint Simon effect, the joint Simon effect was not observed when the participants were explicitly informed that they will be interacting with a computer agent. Additionally, using blindfolded participants, it has been shown that the joint Simon effect does not rely on online information about the co-actor’s actions, but that *a priori* information about the co-actor’s presence is sufficient for the effect to occur (Sebanz et al., 2003; Vlainic et al., 2010). Whilst these studies all suggested the necessity of a human co-actor, several studies suggest that the joint Simon effect is a result of spatial response coding rather than a social influence on action. Guagnano et al. (2010) found that the joint Simon effect occurred when the co-actor was within arm-reach of the participant, but not when the co-actor was distantly seated. Other studies have further demonstrated that a human co-actor is not necessary, by providing salient reference-providing events, such as implementing a Japanese waving cat, a clock, or a metronome (Dolk et al., 2013a), experimenters were able to induce joint Simon effects with non-biological objects. The current experiment, inspired by the referential coding account of the joint Simon effect, which combines aspects of both social and spatial response coding factors, examined the joint Simon effect in both believed biological co-actor, believed computer co-actor, and single Go/Nogo conditions to see when the joint Simon effect occurred and whether there are neurological differences among these different manipulations. We hope to extend the knowledge of the joint Simon effect with both biological and non-biological co-actors compared to the single-actor task.

Behaviorally, in the present experiment, the joint Simon effect was observed in both the biological and computer agent condition, while the single Go/Nogo task did not produce a Simon effect. This finding is consistent with the report by Dolk et al. (2013a) in that a biological agent is not necessary. In addition, these results also show that the joint Simon effect can occur without online spatial coding of the other co-actor. It is worthwhile to note that in our experimental setup, the fMRI session was preceded by a practice joint Simon task outside the scanner with a confederate. It is well-known that the Simon effect is due to the representation of two alternative responses and that previous representations can easily be transferred to subsequent tasks (Ansorge and Wuhr, 2004, 2009; Lugli et al., 2013), thus it is possible that the practice block administrated before the experiment might have introduced carryover effects in the subsequent fMRI tasks. However, this is most likely not the case, since the single Go/Nogo task did not show any significant Simon effects—only the believed human and computer co-actor conditions did.

Turning to the neuroimaging data, by comparing the contrasts of Go trials versus Nogo trials among the three conditions, co-actor conditions (biological and computer) appeared to show increased frontal and visual-parietal activity (including the precentral gyrus, cuneus, precuneus, cingulate gyrus, inferior parietal lobule, middle temporal gyrus, middle occipital gyrus, superior occipital gyrus, etc.) compared to the single actor Go/Nogo condition (see **Figure 3** and **Table 2**). The increased frontal and occipital areas likely reflect differences in stimulus processing when performing with a co-actor (Sebanz et al., 2007). The frontal areas likely reflect increased self-reflective processing during Go trials, and top–down modulation of stimulus valance on Go trials is reflected in the increased activation of the visual association cortex. The inferior parietal lobule and motor areas surrounding the precentral gyrus are part of the mirror neuron system (Molenberghs et al., 2009) and are found to be activated not only when one carries out an action, but also when imagining an action or observing an action carried out in another person (Blakemore and Decety, 2001; Buccino et al., 2001; Ruby and Decety, 2001). It has been suggested that joint tasks act accordingly to the postulate of the ideomotor theory, or common coding theory (Prinz, 1984), where at a certain representational level the planned and perceived actions are functionally equivalent (Sebanz et al., 2003; Tsai et al., 2006). In a similar associative visuomotor task, ventral premotor cortex was involved in the anticipation of a third-person’s response (Ramnani and Miall, 2004). Thus it is not surprising to find increased activation of such areas during the co-actor condition (where anticipation and observation of the co-actor’s response is involved) compared to the single Go/Nogo condition. We suspect that increased Go-Nogo activity (i.e., decreased Nogo-Go activity) in these areas during the co-actor conditions compared to the single Go/Nogo condition, indicates less inhibition during Nogo trials as well as increased representations of their co-actor’s responses. However, these findings contrast with Sebanz et al. (2007) in that they observed decreased activity in the parietal lobule when comparing the contrasts of Go and Nogo trials between a human co-actor condition and the single Go/Nogo condition. The authors suggested that this reflected increased inhibition on Nogo trials when it is the other’s turn. At this moment, however, our experimental design cannot distinguish between action imagery/anticipation/observation and turn-taking.

Next, by comparing the Go-Nogo contrast in the biological and computer co-actor conditions, differences occurred in more frontal areas (including the anterior cingulate gyrus, inferior frontal gyrus, middle frontal gyrus, middle temporal gryus), showing more activation during the computer co-actor conditions. We speculate two possibilities underlying this observation. Firstly, this implies that the contrast of Nogo-Go is greater in the biological co-actor condition. This could indicate greater demands on interference control (i.e., greater response conflict) during Nogo trials in the biological co-actor compared to the computer co-actor condition. This is consistent with the referential coding account (Dolk et al., 2014), in that conflict resolution would be greater when representations of action event are more similar (i.e., in the biological co-actor condition). Second, in a study examining the neural basis of motor imagery (Lorey et al., 2011), while areas of the parieto-premotor network showed positive correlations with perceived vividness, negative correlations were observed primarily in the frontal and temporal areas (including the middle frontal gyrus, inferior frontal gyrus, the superior temporal gyrus, the middle cingulate cortex, the middle part of the temporal gyrus, etc.). Thus, it is possible that the activations in these areas suggest that action monitoring of the co-actor occurs in both co-actor conditions; however, vividness of motor imagery and perhaps self-other integration is more pronounced in the biological co-actor condition.

By investigating the interaction of compatibility and co-action on Go trials, the precuneus, inferior parietal lobule, superior temporal gyrus, and several visual areas were found to show increased compatibility effects in the biological and computer co-actor conditions compared to the single Go/Nogo condition (see **Figure 4** and **Table 3**). This could reflect increased processing of the stimuli in a social context (Sebanz et al., 2007). The parietal lobe is involved in the alerting and orienting networks of attention (Posner and Petersen, 1990; Coull and Nobre, 1998; Raz and Buhle, 2006). We suspect that during co-action, congruent trials receive greater attention and furthermore has an effect of top-down modulation on the visual association cortex.

Most interestingly, there was significant increased activation in the medial prefrontal cortex on congruent trials in the biological co-actor condition compared to the computer co-actor condition. This implies that although the Simon effects were behaviorally similar in these two co-actor conditions, the brain networks supporting the behavior are not totally the same. The medial prefrontal cortex is involved in self-awareness (e.g., Kelley et al., 2002; Goldberg et al., 2006), perspective taking (Vogeley et al., 2004), as well as thinking about the beliefs and intentions of other people (e.g., Mitchell et al., 2005; Amodio and Frith, 2006). This region has also been reported in Sebanz et al. (2007) when contrasting compatibility effects of a biological co-actor and a single Go/Nogo task and in Ramnani and Miall (2004) when comparing brain activity of third-person instruction cues with computer instruction cues. Thus, although behaviorally the joint Simon effect was observed in both co-actor conditions (biological and computer), there are significant differences in the medial prefrontal cortex, which likely reflects a social-cognitive aspect of the joint Simon task when believing a biological co-actor is present. It is also possible that increased activation may also stem from more general processes of action/conflict monitoring. It has been shown that medial prefrontal cortex activation occurs when differentiating the self from intimate others (Heatherton et al., 2006), and according to the referential coding account, the need to differentiate between self- and other- generated events should be more pronounced the more the actor and the co-actor are perceived as similar (i.e., biological co-actor condition). In one study, using gray matter voxel-based morphometry, individual differences of the joint Simon effect were found to correlate negatively with the gray matter of the medial prefrontal cortex (i.e., individuals with greater gray matter showed lesser Simon effects), this probably reflects the role of the medial prefrontal cortex in conflict resolution during joint action (Dolk et al., 2012). Furthermore, using tDCS, cathodal stimulation (inhibitory) of the medial prefrontal cortex led to increased joint Simon effects (Liepelt et al., in press), which suggested the involvement of the medial prefrontal cortex in self-other discrimination during the joint Simon task.

Our results do not fit with the spatial response coding account of the joint Simon effect (Guagnano et al., 2010; Dittrich et al., 2012, 2013), which suggests that the effects strongly result from the spatial location of the co-actor. In our experimental setting, the participant was situated in the scanner room alone with no spatial reference to the co-actor, thus online spatial coding should not occur. Additionally, the joint Simon effect was observed in both believed biological and computer conditions, which also disagrees with Tsai and Brass’s (2007), Tsai et al.’s (2008) view that the effect is tuned to conspecifics. We hypothesize that the joint Simon effect can occur as long as the participant believes that they are interacting with another agent and can represent the agent’s response even oﬄine, regardless of the agent’s identity. Thus, our finding suggests that both biological and non-biological co-actors can induce joint Simon effects; however, additional medial prefrontal cortex is recruited when the co-actor is believed to be a human.

If neither social factors nor online spatial coding was essential to generate the joint Simon effect, then what might have resulted in the joint Simon effect in the current experiment? We suspect that common coding not only occurred between perceived events and intended actions, the participants additionally coded perceived events and anticipated co-actor’s (either biological or computer agent) responses. Ideomotor and common coding theories do not differentiate between social and non-social (i.e., biological and computer) co-actors, but emphasizes only on perception-action linkage. Let us consider the following scenario: we could predict another car driver’s actions through shared visual cues (e.g., traffic lights); but if we were to encounter an automated self-driving car, would we not still anticipate that it will stop at red lights and go at green? Turning back to our experiment, we believe that the joint Simon task is possible with any co-acting agent, during which a response conflict occurs similar to the one that arises when a single person is in charge of both responses. Thus far, our results are compatible with ideomotor-derived theories, including the theory of event-coding (Hommel, 2009), referential coding theory (Dolk et al., 2014), and other extended frameworks (e.g., Prinz, 2015). Lastly, we should note that although neither social nor spatial response coding is essential for generating the joint Simon effect, they may have the potential to modulate how responses are coded and represented.

## Conclusion

The current research investigated the joint-actor and single-actor Go/Nogo tasks in an fMRI scanner. We found that the joint Simon effect can occur oﬄine with both biological and computer co-actors; however, additional medial prefrontal cortex is recruited when acting with a biological agent.

## Conflict of Interest Statement

The authors declare that the research was conducted in the absence of any commercial or financial relationships that could be construed as a potential conflict of interest.
